# Prevention of febrile neutropenia: use of granulocyte colony-stimulating factors

**DOI:** 10.1038/sj.bjc.6605269

**Published:** 2009-09-15

**Authors:** S Kelly, D Wheatley

**Affiliations:** 1 Plymouth Oncology Centre, Derriford Hospital, Plymouth PL6 8DH, UK; 2 Oncology Centre, Royal Cornwall Hospital, Truro, Cornwall TR1 3LJ, UK

**Keywords:** dose intensity, febrile neutropenia, G-CSF, guidelines, prophylaxis

## Abstract

There is good evidence to suggest that dose intensity is important when considering the effectiveness of adjuvant chemotherapy in patients with breast cancer. However, the development of chemotherapy-induced febrile neutropenia can lead to reduction in dose intensity and other treatment modifications, which may negatively affect patient outcomes. Febrile neutropenia can be prevented by the use of primary prophylactic treatment, notably with granulocyte colony-stimulating factors. This practice is supported by international guidelines, all of which recommend that primary prophylaxis with granulocyte colony-stimulating factors should be used with chemotherapy where the risk of febrile neutropenia is 20% or greater.

Use of a chemotherapy regimen containing a taxane or an anthracycline in the adjuvant treatment of patients with breast cancer has been shown to have benefits in terms of both time to disease progression and overall survival (Martin *et al*, 2005; Peto *et al*, 2007). However, such regimens are also associated with the potentially serious side effects of febrile neutropenia (FN) and neutropenic sepsis (Martin *et al*, 2005; Peto *et al*, 2007). FN is not only a major risk factor for morbidity and mortality in patients with cancer (Aapro *et al*, 2006; Herbst *et al*, 2009), but its development can also lead to a decision to reduce the chemotherapy dose and delay subsequent treatment cycles (Aapro *et al*, 2006). Such treatment modifications are a particular concern when chemotherapy is given with curative intent (Aapro *et al*, 2006), and hence the importance of FN prevention.

FN can be prevented through the prophylactic use of haematopoietic cell growth factors (e.g., granulocyte colony-stimulating factors, G-CSF) – a strategy supported by current guidelines for patients deemed to be at high risk of FN (Aapro *et al*, 2006; Smith *et al*, 2006; Segal *et al*, 2008). The indications for prophylactic administration of G-CSF are based on various risk factors, including the degree of myelosuppression associated with the chemotherapy regimen, and specific patient characteristics (Aapro *et al*, 2006; Smith *et al*, 2006).

The management of serious neutropenic events is described elsewhere in this supplement (Cameron, 2009), as is the antibiotic-based prophylaxis of FN (Cullen and Baijal, 2009). This article focuses on the potential consequences of modifying chemotherapy dose density in response to FN, and the alternative approach – primary prevention of FN through the prophylactic use of G-CSF.

## FN morbidity and mortality

Chemotherapy-induced neutropenia is a major risk factor for infection-related morbidity (Aapro *et al*, 2006), including fungal infections, Gram-negative sepsis, pneumonia and other lung disease, cerebrovascular disease and disorders of the liver and kidney (Kuderer *et al*, 2006).

The mortality rates associated with FN range from 2 to 21% (Herbst *et al*, 2009), and the risk of death is increased by various factors, including patient characteristics, type of malignancy, presence of comorbidities and infectious complications (Kuderer *et al*, 2006).

## Dose intensity

Development of FN and neutropenic sepsis leads to significant morbidity and mortality, and is commonly regarded as an indication for a dose reduction or cycle delay among patients receiving chemotherapy (Aapro *et al*, 2006). However, there is evidence that such changes to chemotherapy dose intensity (DI, dose delivered divided by the overall duration of treatment) can have a negative effect on treatment outcomes (Wood *et al*, 1994; Bonadonna *et al*, 1995; Leonard *et al*, 2003; Bonneterre *et al*, 2005; Chirivella *et al*, 2006).

A retrospective analysis of patients undergoing adjuvant treatment with classical 28-day CMF (cyclophosphamide, methotrexate and fluorouracil) showed a major survival benefit among those who received 85% or more of the intended dose (Bonadonna *et al*, 1995). Delivery of 65–85% of the planned dose was associated with some benefit, but patients who received less than 65% fared no better than did those who received no chemotherapy at all.

Another study compared three regimens of FAC (fluorouracil, doxorubicin and cyclophosphamide) that differed in their DI: moderate-DI FAC (fluorouracil 40 mg m^–2^ on days 1 and 8, and doxorubicin 40 mg m^–2^ and cyclophosphamide 400 mg m^–2^ on day 1 for six cycles), higher-DI FAC (the same total dose as moderate-DI FAC but delivered in just four cycles) and lower-DI FAC (half the total dose of high-DI and moderate-DI FAC, delivered in four cycles) (Wood *et al*, 1994). After 3 years, there was no difference between the moderate-DI and higher-DI groups in terms of disease-free and overall survival, but both outcomes were significantly poorer in the lower-DI group. The authors propose a threshold effect for chemotherapy delivery, that is, a DI below which the benefit is significantly reduced (Wood *et al*, 1994).

An association between increased DI and improved outcomes has also been reported by the French Adjuvant Study Group (Bonneterre *et al*, 2005), which compared two regimens of FEC. Patients receiving adjuvant chemotherapy for breast cancer were given either FEC 50 (fluorouracil 500 mg m^–2^, epirubicin 50 mg m^–2^ and cyclophosphamide 500 mg m^–2^) or FEC 100 (as FEC 50 but with the epirubicin dose increased to 100 mg m^–2^). At 10 years, there was a significant benefit in favour of FEC 100 in the rates of disease-free survival (FEC 50, 45.3% *vs* FEC 100, 50.7%, *P*=0.036) and overall survival (50.0 *vs* 54.8%, respectively, *P*=0.038).

Looking specifically at the reduced DI brought about by dose delay, a retrospective analysis of the records of 793 patients who received adjuvant chemotherapy for breast cancer between 1980 and 2000 has shown that, after a median follow-up of 10 years, patients exposed to more than two cycle delays had poorer event-free and overall survival, compared with those whose treatment was delivered on time (Chirivella *et al*, 2006). In this study, treatment delay was defined as any cycle being delivered more than 3 days later than planned, the whole course being completed more than 15 days later than planned or a delivery of less than 95% of the planned DI.

The association between the development of severe neutropenia and reduced DI has been investigated in an audit of 422 patients with breast cancer who received adjuvant chemotherapy (mainly CMF or anthracycline-based) in 15 UK centres; 29% of the patients had at least one neutropenic event (defined as hospitalisation because of FN, a dose delay of 7 days or more because of neutropenia, and/or a dose reduction of 15% or more because of neutropenia), and 17% of the patients received less than 85% of the planned total dose of their regimen (Leonard *et al*, 2003). Patients who experienced a neutropenic event received a significantly lower DI than those who did not. Around 40% of patients undergoing CMF-based chemotherapy and 32% of patients undergoing anthracycline-based chemotherapy who experienced a neutropenic event received less than 85% of their intended dose. Interestingly, only 5.2% of patients in the study received haematopoietic cell growth factors at any time during their treatment.

## Choice of chemotherapy

There are many factors to consider when choosing a suitable chemotherapy regimen for an individual patient, and little research to show why some patients who seem suitable for a particular regimen do not receive it. However, there are anecdotal reports either that patients may decline effective chemotherapy or that physicians may be unwilling to prescribe it, because of concerns about toxicity – even when the likely side effects can be effectively prevented or managed. For example, the findings of a survey of 50 UK oncologists, conducted in 2008, suggested that FN was a deterrent to the prescription of docetaxel, doxorubicin and cyclophosphamide (TAC) for patients with operable node-positive early-stage breast cancer. Only 58% of eligible patients were considered for the regimen, and only 32% of the eligible population actually received it (Oncologist Breast Cancer Study, 2008). The respondents cited concern about FN as the major barrier to prescribing TAC. Only 24% of the oncologists used G-CSF for primary prophylaxis of FN.

Gounaris *et al* (2008) have shown that a patient's age can also be a major factor affecting the decision to use particular chemotherapy regimens. In their study, the mean ages of patients with node-positive breast cancer prescribed taxane-based chemotherapy, anthracycline-based chemotherapy or no chemotherapy were 52.7, 59.4 and 73.2 years, respectively. Whether age *per se* should affect the choice of chemotherapy will be discussed in detail later.

## Guidelines on prevention of FN

Chemotherapy dose reduction/delay is not the only strategy available for reducing FN-related morbidity and mortality. Another option is FN prevention through prophylactic treatment with G-CSF, granulocyte–macrophage colony-stimulating factors (GM–CSF) and/or antibiotics (Herbst *et al*, 2009).

Haematopoietic cell growth factors stimulate the proliferation and survival of neutrophils and their precursors, and thereby reduce the severity and duration of chemotherapy-induced neutropenia and FN (Ozer *et al*, 2000; Komrokji and Lyman, 2004; Segal *et al*, 2008).

**Figure 1:**
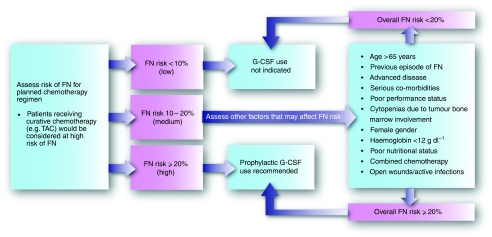
Algorithm for FN prevention in patients receiving chemotherapy, based on guidelines by the European Organization for Research and Treatment of Cancer (Aapro *et al*, 2006) and the American Society of Clinical Oncology (Smith *et al*, 2006).

Indications for the primary prevention of FN through the use of G-CSFs for patients undergoing chemotherapy have been issued by three well-respected oncology organisations – the American Society of Clinical Oncology (Smith *et al*, 2006), the European Organization for Research and Treatment of Cancer (Aapro *et al*, 2006) and the National Comprehensive Cancer Network (Segal *et al*, 2008). They all broadly agree that any patient with an FN risk greater than 20% should receive primary prophylaxis with G-CSF with each cycle of chemotherapy (Figure 1). In some instances, the chemotherapy regimen itself carries an FN risk that exceeds this threshold (Aapro *et al*, 2006). If the FN risk associated with the regimen is 10–20%, the physician should consider whether patient factors such as age, advanced disease or comorbidities take the overall risk beyond 20%. If the chemotherapy regimen is considered to present an FN risk of less than 10%, primary prophylaxis with G-CSF should not be offered routinely – unless there is thought to be a huge risk of serious FN complications, such as death. However, it is highly likely that G-CSF primary prophylaxis is not always used routinely in clinical practice, despite these clear, evidence-based guidelines.

## Evidence supporting primary prophylaxis of FN

In a meta-analysis of 17 randomised controlled trials involving more than 3400 patients undergoing chemotherapy, infection-related mortality was 1.5% in those who received primary prophylaxis with G-CSF, compared with 2.8% in controls (relative risk (RR)=0.055) (Kuderer *et al*, 2007). The rate of early morbidity fell from 3.4% in controls to 2.7% in patients receiving G-CSF. FN occurred once or more in 39.5% of controls and 22.4% of G-CSF-treated patients (RR=0.54). In 10 trials that monitored DI, the meta-analysis found that all G-CSF recipients had a DI higher than 90%. By contrast, DI in the control arms was lower than 85% in six of the 10 trials.

The efficacy of pegfilgrastim, a prolonged-action pegylated form of G-CSF, in the prevention of FN associated with chemotherapy based on docetaxel (100 mg m^–2^) in breast cancer was assessed in a study of 928 patients with metastatic breast cancer randomised to either placebo or pegfilgrastim (Vogel *et al*, 2005). Compared with the placebo group, the pegfilgrastim-treated patients had a significantly lower rate of FN (17 *vs* 1%, respectively, *P*<0.001), FN-related hospital admissions (14 *vs* 1%, *P*<0.001) and use of intravenous antibiotics for the management of infections (10 *vs* 2%, *P*<0.001). During cycle 1 of chemotherapy, 11% of patients who received placebo developed FN (i.e., two-thirds of all episodes of FN in the placebo group), compared with 1% of pegfilgrastim recipients. As patients who were given placebo and developed FN were then allowed to receive pegfilgrastim during subsequent chemotherapy cycles, the DI was not significantly different between the two groups.

The international guidelines are supported further by recent results from the GeparTrio trial, in which patients with breast cancer who received neoadjuvant TAC were given the following prophylactic treatments: the antibiotic ciprofloxacin (500 mg twice daily on days 5–14), non-pegylated G-CSF (5 *μ*g kg^–1^ day^–1^ filgrastim or 150 *μ*g m^–2^ day^–1^ lenograstim on days 5–10), pegfilgrastim (6 mg on day 2) or a combination of the pegfilgrastim and the ciprofloxacin schedules (von Minckwitz *et al*, 2008). Both FN and grade 4 neutropenia were significantly more frequent in patients who received either ciprofloxacin alone or non-pegylated G-CSF, compared with those given pegfilgrastim with or without ciprofloxacin. Indeed, no patients receiving the combination of pegfilgrastim and ciprofloxacin developed FN during chemotherapy cycle 1.

The combined approach also proved beneficial in a study by Gounaris *et al* (2008). In the study, patients given the complete primary prophylaxis schedule of lenograstim (263 μg subcutaneously on days 4–10) and ciprofloxacin (500 mg twice daily on days 5–14) had an FN rate of 0.8, and 7% needed a modification to their chemotherapy regimen (Gounaris *et al*, 2008). By contrast, when prophylaxis was inadequate (usually because G-CSF was started later than day 5), the risk of FN was 15, and 36% of patients required modification to their chemotherapy.

## FN prophylaxis in clinical practice

Physicians need to be aware of the FN risk associated with each regimen that they prescribe and take into account the patient-related factors that increase that risk. Primary prophylaxis with G-CSF should always be offered when the risk of FN is 20% or greater. Prophylactic use of antibiotics should also be considered, as discussed elsewhere in this supplement by Cullen and Baijal.

G-CSF treatment should start within 24–72 h of the chemotherapy dose, and be administered for long enough to allow adequate neutrophil recovery. Treatment schedules associated with three commonly used G-CSF formulations are summarised in Table 1. Pegfilgrastim is administered just once per cycle, whereas shorter acting products are used daily. The duration of use of daily G-CSF is variable. Trial evidence has shown that FN rates are lower in patients who receive pegfilgrastim than in those who receive 11 days of daily G-CSF (von Minckwitz *et al*, 2008), which suggests that the common practice of giving daily G-CSF for 4–7 days may provide suboptimal prophylaxis.

### Risk assessment

The management of chemotherapy-induced FN is gaining increasing attention as evidence accumulates to indicate that a proactive, preventive approach may improve the delivery of care and patient outcomes. Both chemotherapy and patient-related factors are important when assessing which chemotherapy regimen and supportive treatments to give to a particular patient.

The risks associated with both the prescribed chemotherapy regimen and patient factors (Figure 1) must be assessed before commencing chemotherapy and before delivering each subsequent cycle (Aapro *et al*, 2006).

The choice of chemotherapy is generally the main risk factor for FN, and if two different regimens are believed to offer equal efficacy to the patient, the regimen with the lower risk of FN should be chosen. However, looking specifically at patients with breast cancer, it is well documented that taxane-containing regimens are significantly more effective than those without taxanes (Peto *et al*, 2007), at the cost of increased FN rates (Bria *et al*, 2005).

Several patient risk factors are associated with development of FN. There is evidence from well-designed trials showing that FN is more likely to develop in patients older than 65 years and in those with advanced disease, a previous history of FN and low performance status (Aapro *et al*, 2006). There is also evidence suggesting other FN risk factors, including female sex, haemoglobin below 12 g dl^–1^, cardiovascular disease, renal disease and abnormal liver function tests, in particular raised bilirubin (Aapro *et al*, 2006; Lyman *et al*, 2007). Careful assessment of all the treatment and patient factors should be formulated, and the guidelines closely adhered to.

### Elderly patients

Elderly patients account for only a small proportion of the population with early-stage breast cancer (prevalence approaching 7% among the over-70 s) (Louwman *et al*, 2007). This group is worth mentioning, however, as elderly patients present particular challenges, including reduced tolerance to chemotherapy (Wedding *et al*, 2007) and greater susceptibility to the development of FN (Lyman and Dale, 2003). Importantly, with careful patient selection, chemotherapy can be effective and tolerable in older as well as younger individuals.

It is important to note that chronological age, by itself, is not a reliable indicator of life expectancy, functional reserve or the risk of treatment complications (Wedding *et al*, 2007). Elderly patients require a comprehensive geriatric assessment, looking at function, comorbidities, nutritional status, cognition, emotional evaluation and socioeconomic issues (Wedding *et al*, 2007).

A systematic review of the literature has provided evidence to support the use of haematopoietic growth factors in elderly people, to reduce the risk of neutropenic events and the need for reduced DI (Lyman and Dale, 2003), but specific evidence for the benefits of primary prophylaxis of FN in elderly breast cancer patients is currently lacking. Further studies are needed.

## Role of secondary prophylaxis with G-CSF

Patients with a high risk of FN (over 20%) should be offered primary prophylaxis with G-CSF, in accordance with guidelines (Aapro *et al*, 2006; Smith *et al*, 2006; Segal *et al*, 2008). However, a predicted low risk (under 10%) of FN is no guarantee that the side effect will not occur. Secondary prophylaxis during subsequent cycles is therefore an option if a low-risk patient develops FN during chemotherapy.

There are no prospective studies of the value of secondary G-CSF prophylaxis (Hupperets and Tjan-Heijnen, 2006). After patients in a pivotal G-CSF trial were allowed to switch from placebo to G-CSF if they developed FN in the first cycle (Crawford *et al*, 1991), the FN rate fell from 100% in cycle 1 to 23% in cycle 2. However, definite conclusions on the efficacy of secondary prophylaxis cannot be drawn from these observations because many trials report a decline in the incidence of FN in later cycles without administering prophylaxis (Hupperets and Tjan-Heijnen, 2006).

Secondary prophylaxis should be offered if a patient develops FN, despite being in a low FN risk category, and it is judged important to maintain their DI. However, dose reduction or delay may be appropriate for some patients such as, for example, those receiving palliative chemotherapy and those who develop grade 3/4 non-haematological toxicities that cannot be attributable to FN. If a patient is likely to develop a serious complication resulting from FN, such as death, then primary prophylaxis should be considered.

## Conclusion

As well as being associated with morbidity and mortality (Aapro *et al*, 2006; Kuderer *et al*, 2006), chemotherapy-induced FN can also lead to treatment modifications and poorer outcomes for patients – a particular concern when chemotherapy is being given with curative intent (Aapro *et al*, 2006). However, primary prophylaxis of FN, using G-CSF, can reduce the risk of FN developing in the first place (Aapro *et al*, 2006; Smith *et al*, 2006; Segal *et al*, 2008). Regimen-based and patient-based indications for its use are set out in international guidelines (Aapro *et al*, 2006; Smith *et al*, 2006; Segal *et al*, 2008). Patients whose risk of chemotherapy-induced FN is 20% or greater should be given primary prophylactic G-CSF. If a patient develops FN during a less intensive regimen, and DI needs to be maintained, secondary prophylactic G-CSF should be strongly considered for the support of subsequent treatment cycles.

## Figures and Tables

**Table 1:** G-CSF treatment schedules

**Formulation**	**Route**	**Dose**	**Frequency**	**Duration**
Lenograstim	Subcutaneous	150 *μ*g m^–2^	Daily	28 consecutive days
Filgrastim	Subcutaneous	5 *μ*g kg^–1^	Daily	Up to 38 days
Pegfilgrastim	Subcutaneous	6 mg^–1^	Once per cycle	Once per cycle
